# A Comparison between Metaheuristics as Strategies for Minimizing Cyclic Instability in Ambient Intelligence

**DOI:** 10.3390/s120810990

**Published:** 2012-08-08

**Authors:** Leoncio A. Romero, Victor Zamudio, Rosario Baltazar, Efren Mezura, Marco Sotelo, Vic Callaghan

**Affiliations:** 1 Division of Research and Postgraduate Studies, Leon Institute of Technology, Leon, Guanajuato 37290, Mexico; E-Mails: charobalmx@yahoo.com.mx (R.B.); masotelof@gmail.com (M.S.); 2 Laboratorio Nacional de Informatica Avanzada, Xalapa, Veracruz 91000, Mexico; E-Mail: emezura@lania.mx; 3 School of Computer Science and Electronic Engineering, University of Essex, Wivenhoe Park CO4 3SQ, UK; E-Mail: vic@essex.ac.uk

**Keywords:** cyclic instability, ambient intelligence, locking

## Abstract

In this paper we present a comparison between six novel approaches to the fundamental problem of cyclic instability in Ambient Intelligence. These approaches are based on different optimization algorithms, Particle Swarm Optimization (PSO), Bee Swarm Optimization (BSO), micro Particle Swarm Optimization (*μ*-PSO), Artificial Immune System (AIS), Genetic Algorithm (GA) and Mutual Information Maximization for Input Clustering (MIMIC). In order to be able to use these algorithms, we introduced the concept of *Average Cumulative Oscillation* (ACO), which enabled us to measure the average behavior of the system. This approach has the advantage that it does not need to analyze the topological properties of the system, in particular the loops, which can be computationally expensive. In order to test these algorithms we used the well-known discrete system called the Game of Life for 9, 25, 49 and 289 agents. It was found that PSO and *μ*-PSO have the best performance in terms of the number of agents locked. These results were confirmed using the Wilcoxon Signed Rank Test. This novel and successful approach is very promising and can be used to remove instabilities in real scenarios with a large number of agents (including nomadic agents) and complex interactions and dependencies among them.

## 1. Introduction

Any computer system can have errors and Ambient Intelligence is not exempt from them. Cyclical instability is a fundamental problem characterized by the presence of unexpected oscillations caused by the interaction of the rules governing the agents involved [1–4].

The problem of cyclical instability in Ambient Intelligence is a problem that has received little attention by the designers of intelligent environments [2,5]. However in order to achieve the vision of AmI this problem must be solved.

In the literature there are several strategies reported based on analyzing the connectivity among the agents due to their rules. The first one the Instability Prevention System INPRES is based on analyzing the topological properties of the Interaction Network. The Interaction Network is the digraph associated to the system and captures the dependencies of the rules between agents. INPRES finds the loops and locks a subset of agents on a loop, preventing them to change their state [1–4]. INPRES has been tested successfully in system with low density of interconnections and static rules (nomadic devices and time variant rules are not allowed). However when the number of agents involved in the system increases (with high dependencies among them) or when the agents are nomadic, the approach suggested by INPRES is not practical, due to the computational cost.

Additionally Action Selection Algorithms map the relationship between agents, rules and selection algorithms, into a simple linear system [6]. However this approach has not been tested in real or simulated scenarios nor nomadic agents.

The approach presented in this paper translates the problem of cyclic instability into a problem of intelligent optimization, moving from exhaustive search techniques to metaheuristics searching.

In this paper we compare the results of *Particle Swarm Optimization* (PSO), *Bee Swarm Optimization* (BSO), *micro Particle Swarm Optimization* (*μ*-PSO), *Artificial Immune System* (AIS), *Genetic Algorithm* (GA) and *Mutual Information Maximization for Input Clustering* (MIMIC) when applied to the problem of cyclic instability. These algorithms find a good set of agents to be locked, in order to minimize the oscillatory behavior of the system. This approach has the advantage that there is no need to analyze the dependencies of the rules of the agents (as in the case of INPRES). We used the game of life [7] to test this approach, where each cell represents an agent of the system.

## 2. Cyclic Instability in Intelligent Environments

The scenarios of intelligent environments are governed by a set of rules, which are directly involved in the unstable behavior, and can lead the system to multiple changes over a period of time. These changes can cause interference with other devices, or unwanted behavior [1–4].

The state of a system *s(t)* is defined as the logarithm base 10 of the binary vectors of the agents. A turned-on agent was represented with 1 while a shut-down agent will be represented with 0. The representation of the state of the system is shown in Equation (1):

(1) s(t)=log(s)

where *s*(*t*) is the state of the system at time *t*, and *s* is base-10 representation of the binary number of the state of the system.

## 3. Optimization Algorithms

### 3.1. Particle Swarm Optimization

The Particle Swarm Optimization (PSO) algorithm was proposed by Kennedy and Everhart [8,9]. It is based on the choreography of a flock of birds [8–13], wing a social metaphor [14] where each individual brings their knowledge to get a better solution. There are three factors that influence the change of the particle state or behavior:

- Knowledge of the environment or adaptation is the importance given to past experiences.
- Experience or local memory is the local importance given to best result found.
- The experience of its neighbors or neighborhood memory is the importance given to the best result achieved by their neighbors.

The basic PSO algorithm [9] uses two equations. Equation (2), which is used to find the velocity, describes the size and direction of the step that will be taken by the particles and is based on the knowledge achieved until that moment.

(2) $${\text{v}}_{\text{i}}={\text{wv}}_{\text{i}}+{\text{c}}_{1}{\text{r}}_{1}({\text{l Best}}_{\text{i}}-{\text{x}}_{\text{i}})+{\text{c}}_{2}{\text{r}}_{2}(\text{g Best}-{\text{x}}_{\text{i}})$$

where:

*v_i_* is the velocity of the i-th particle, *i* = 1, 2 …, *N*,
*N* is the number of the population,
*w* is the environment adjustment factor,
*c*_1_ is the memory factor of neighborhood,
*c*_2_ is memory factor,
*r*_1_ and *r*_2_ are random numbers in range [0, 1],
*lBest* is the best local particle founded for the i-th particle,
*gBest* is the best general particle founded until that moment for all particles.

Equation (3) updates the current position of the particle to the new position using the result of the velocity equation.

(3) $${\text{x}}_{\text{i}}={\text{x}}_{\text{i}}+{\text{v}}_{\text{i}}$$

where *x_i_* is the position of the i-th particle.

The PSO algorithm [9] is shown in the Algorithm 1:

**Algorithm 1** PSO Algorithm.
**Data:**
*P* ∈ [3, 6] (number of particles), *c*_1_ ∈ *R, c*_2_ ∈ *R, w* ∈ [0, 1], *G* (maximum allowed function evaluations).**Result:**
*GBest* (best solution found)
Initialize particles ' position and velocity randomly;For *g* = 1 to *G* doRecalculate best particles position *gBest*Select the local best position *lBest*For each X*ig, i* = 1, …, *P do*Recalculate particle speedRecalculate particle position


### 3.2. Binary PSO

Binary PSO [13,14] was designed to work in binary spaces. Binary PSO select the *lBest* and *gBest* particles in the same way as PSO. The main difference between binary PSO and normal PSO are the equations that are used to update the particle velocity and position. The equation for updating the velocity is based on probabilities but these probabilities must be in the range [0, 1]. A mapping is established for all real values of velocity to the range [0, 1]. The normalization Equation (4) used here is a sigmoid function.

(4) $${\text{v}}_{\text{ij}}(\text{t})=\text{sigmoid}\;({\text{v}}_{\text{ij}}(\text{t}))=\frac{1}{1+e^{-{\text{v}}_{\text{ij}}(t)}}$$

and Equation (5) is used to update the new particle position.

(5) $${\text{x}}_{\text{ij}}(\text{t}+1)=\{\begin{array}{c} 1{\text{ifr}}_{\text{ij}}<\text{sigmoid}\;({\text{v}}_{\text{ij}}(\text{t}+1))\\ 0\;\text{in other case} \end{array}$$

where *r_ij_* is a random vector with uniform values in the range [0, 1].

### 3.3. Micro PSO

*μ*-PSO algorithm [15,16] is a modification made to the original PSO algorithm in order to work with small populations (See Algorithm 2). This algorithm use replacement and mutation to be included in the original PSO algorithm, and allow to the algorithm avoid local optima.

### 3.4. Bee Swarm Optimization

BSO algorithm [14] is based on PSO and Bee algorithm (See Algorithm 3). It uses a local search algorithm to intensify the search. This algorithm was proposed by Sotelo [14], where the changes made to PSO allowed finding a new best Global in the current population.



**Algorithm 2**
*μ*-PSO Algorithm.
**Data:**
*P* ∈ [3, 6] (population size). *R* > 0 (Replacement generation), *N* > 0 (Number of restart particles), *M* ∈ [0, 1] (Mutation Rate;, *c*_1_ ∈ *R, c*_2_ ∈ *R, w* ∈ [0, 1], *Neighborhoods* > 0, *MaxFes* (maximum allowed function evaluations).**Result:**
*GBest* (best solution found)
Initialize particles' position, velocity and neighborhood randomly;*Set cont* = 1 *and G* = *MaxFes/P**For g* = 1 *to G do**If* (*cont* == *R*)Reinitialization of*N worst particles**Set cont* = 1Recalculate best particles position *gBest*Select the local best position in the neighborhood *lBest*For each *Xig, i* = 1, …, *P do*Recalculate particle speedRecalculate particle positionPerform mutation to each particle with a probability of *P*(*M*);*Set cont* = *cont* + 1


**Algorithm 3** BSO Intensifier.

*Generate two new bees looking around to gBest*.*Evaluate the fitness for generated bees in the D variables*.*Select the generated bee with best fitness*.If the fitness of selected bee is better than gBest*Set the value and position of gBest as the bee*.*Otherwise increases gBest age*.


Another variant of this algorithm consists in applying the steps in each comparison of a bee with the local best.

In the future we will refer to BSO algorithm with the global best enhancer as BSO1, while BSO algorithm with local the best enhancer will be known as BSO2.

### 3.5. Artificial Immune System

The Artificial Immune System (AIS) [17] is a metaheuristic based on the Immune System behavior of living things [14], particularly of mammals [17].

One of the main functions of the immune system is to keep the body healthy. A variety of harmful microorganisms (called pathogens) can invade the body. Antigens are molecules that are expressed on the surface of pathogens that can be recognized by the immune system and are also able to initiate the immune response to eliminate the pathogen cells [17].

Artificial immune systems have various types of model (See Algorithm 4). In our case we use the one that implements the clonal selection algorithm, which emulates the process by which the immune system, in the presence of a specific antigen, stimulates only those lymphocytes that are more similar [17].

The steps of the algorithm are described below:

**Algorithm 4** Artificial Immune System Algorithm.
**Data:**
*tam* number of antibodies, *n* antibodies to select, *d* number of new antibodies, *B* multiplication factor Beta, *Iterations* number of executions of the metaheuristic.**Result:**
*sol* antibodies
*sol* ← *Generate(tam)* {Initiates the population according to the initial population selected and gains the fitness of each of their antibodies}*Sort(sol)* {Sort antibodies based in fitness}**For** *i* = *1* to *Iterations* **do***sol*-best ← *Select(sol,n)* {Select the *n* best antibodies}*sol-copied*← *Copy(sol-best,B)* {Copy *B* times the best antibodies}*sol-hyper* ← *Hypermutability(sol-copied)* {Hypermutability the copied antibodies}*sol-best* ← *Select(sol-hyper, n)* {Select the *n* best antibodies obtained by hypermutability}*sol* ← *sol*+*sol-best* {Add the best antibodies obtained to antibodies}*sol-new* ← *Generate(d)* {*d* new antibodies are generated according to the initial population selected}*sol* ← *Replace(Sol, sol-new)* {Replace d worst antibodies of population with the newest generated antibodies}*Sort (sol)* {Sort antibodies based on fitness}*Resize(sol, tam)* {Resize the number of antibodies that have the same number when the metaheuristic begin}


### 3.6. Genetic Algorithm

The genetic algorithm (GA) [18] is a search technique proposed by John Holland based on the theory of evolution by Darwin [18–20]. This technique is based on the selection mechanisms that nature uses, according to which the fittest individuals in a population are those who survive, to adapt more easily to changes in their environment.

A fairly comprehensive definition of a genetic algorithm is proposed by John Koza [21]:

“It is a highly parallel mathematical algorithm that transforms a set of individual mathematical objects with respect to time using operations patterned according to the Darwinian principle of reproduction and survival of the fittest and after naturally have arisen from a series of genetic operations from which highlights the sexual recombination. Each of these mathematical objects is usually a string of characters (letters or numbers) of fixed length that fits the model of chains of chromosomes and is associated with a certain mathematical function that reflects their ability”.

The GA seeks solutions in the space of a function through simple evolution. In general, the individual fitness of a population tends to reproduce and survive to the next generation, thus improving the next generation. Either way, inferior individuals can, with a certain probability, survive and reproduce. In Algorithm 5, a genetic algorithm is presented in a summary form [19].


**Algorithm 5** Genetic Algorithm.
**Data:**
*t* (population size), *G* (maximum allowed function evaluations).**Result:**
*Best Individual* (Best Individual of last population).
*P* ← *Initialize-population(t)* {Generate (randomly) an initial population }*Evaluate(P)* {Calculate the fitness of each individual}*For g* = *1 to G do**P* ' ← *Select(P)* {Choose the best individuals in the population and pass them to the next generation}*P* ' ← *Cross(P)* {Cross population to generate the rest of next population}*P* ' ← *Mutation(P*″) {Mutate one individual of population randomly chosen}*Evaluate(P* ') {Calculate the fitness of each individual of new population}*P* ← *P* ') {Replace the old population with new population}


#### 3.6.1. Clones and Scouts

In order to increase performance of the GA the concept of clones and explorers is taken [22]. A clone is one individual whose fitness is equal to the best individual fitness. When it reaches a certain percentage of clones a percentage of the worst individuals in the population is then mutated. Mutated individuals are named scouts. The application of clones and explorers is in addition to the mutation carried by the GA generation.

The algorithm to implement and explorers clones is shown in the Algorithm 6 [22].


**Algorithm 6** Clones and Scouts Algorithm.

*Define PC* (*Clones percentage*)Count the number of individuals who have the same fitness value than the best individual of current population and calculate*PAC (*Accumulative Percentage of Clones)*Define PE (*Scouts percentage)*If PAC* ≥ *PC*Select the worst*N* (*PE*) individuals of the current population, where *N* is the size of the population of individualsMutate selected individuals


## 4. Mutual Information Maximization for Input Clustering

The Mutual Information Maximization for Input Clustering (MIMIC) [14,23–25] is part of the algorithms known as EDAs (Estimation of Distribution Algorithms). These algorithms aim to get the probabilistic distribution of a population based on a set of samples, searching for the permutation associated to the lowest value of the Kullback–Leibler divergence (see Equation (6)). This value is used to calculate the similarity between two different sets of samples:

(6) $${\text{H}}_{\text{l}}^{\pi }={\text{h}}_{\text{l}}({\text{X}}_{\text{in}})+\sum_{\text{j}=1}^{\text{n}-1}{\text{h}}_{\text{l}}({\text{x}}_{\text{ij}}|{\text{x}}_{\text{ij}+1})$$

where:

*h* (*x*) = − Σ*_x_ p* (*X* = *x*) log *p* (*X* = *x*) is Shannon's entropy of *X* variable
*h* (*X*|*Y*) = − Σ*_x_ p* (*X*|*Y*) log *p* (*Y* = *y*), where
*h* (*X*|*Y* = *y*) = − Σ*_X_ p* (*X* = *x*|*Y* = *y*) log (*X* = *x*|*Y* = *y*), is the *X* entropy given *Y*.

This algorithm suppose that the different variables have a bivariate dependency described by Equation (7).

(7) $${\text{p}}_{\text{l}}^{\pi }(\text{x})={\text{p}}_{\text{l}}({\text{x}}_{\text{i}1}|{\text{x}}_{\text{i}2})▪{\text{p}}_{\text{l}}({\text{x}}_{\text{i}2}|{\text{x}}_{\text{i}3})\cdot \ldots {\text{p}}_{\text{l}}({\text{x}}_{\text{in}-1}|{\text{x}}_{\text{in}}){\text{p}}_{\text{l}}(x_{\text{in}})$$

where *π* = (*i*1, *i*2, …, *in*)is an index permutation.

The algorithm can be seen in Algorithm 7:

**Algorithm 7** MIMIC Algorithm.

Initialize a population (array) of individuals with random values on d dimensions in the problem spaceSelect a subpopulation through a selection method*Calculate Shannon*'*s entropy for each variable*.*Generate a permutation* ${\text{p}}_{\text{l}}^{\pi }(\text{x}).$*Choose variable with lowest entropy*.*For the next variables choose i_k_* = *argmin_j_h_l_* (*X_j_*|*X_ik_*_+1_) *Where j* ≠ *i_k_*+*_i_*, …,*in**Sample the new population using the generated permutation* $p_{l}^{\pi }(x).$*Loop until a criterion is met*.


## 5. Test Instances

The Game of Life [7] was used as test instance for the problem of cyclical instability affecting intelligent environments. This is because it has been shown in many cases that the Game of Life system exhibits oscillatory behavior in spite of the few rules used for this. In addition the environment is extremely simple and may take from a few to a large number of agents acting within the system.

The game of life is a cellular automata created by John Conway [7]. This is a set of cells which, based on a set of simple rules, can live, die or multiply. Depending on the initial conditions, the cells form different patterns during the course of the game. The rules of the game are as follows [7] :

1. *Survival*: if a cell is in state 1 and has 2 or 3 neighbors in state 1, then the cell remains in state 1
2. *Birth*: if a cell is in state 0 and has exactly 3 neighbors in state 1, the next time step the cell goes to state 1.
3. *Deaths*: a cell in state 1 goes to state 0 if it has 0 or 1 neighbors, or if it has 4 or more neighbors.

From the game of life, we have examples of oscillating behavior. From these we take the follow configurations as benchmark.

**Figure 1.**
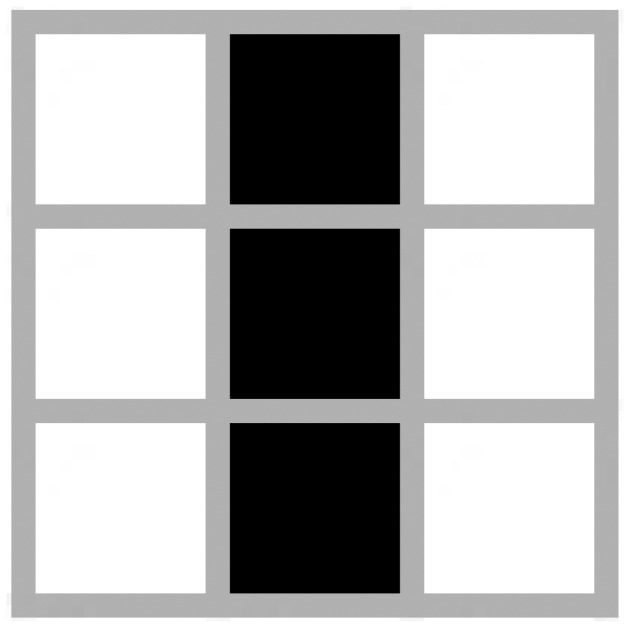
Blinker.

Figure 1 presents the simplest known oscillator in the Game of Life called Blinker. This oscillator with 3 alive cells fits neatly into a 3 × 3 grid with 9 potential agents in this scenario.

**Figure 2.**
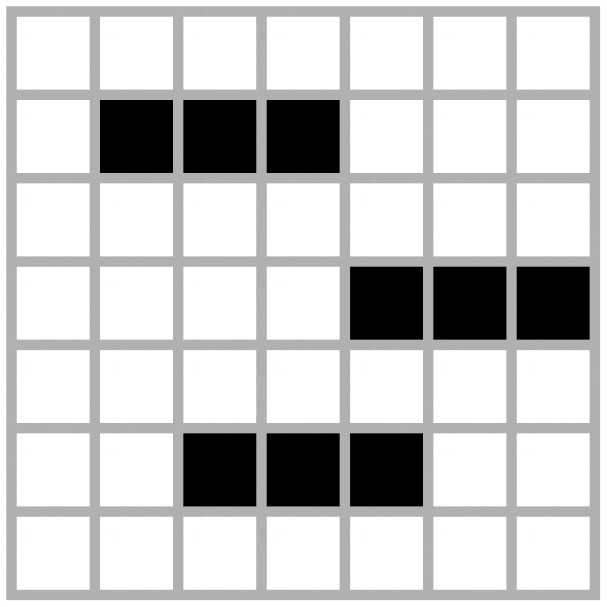
Non-oscillating.

The settings in Figure 2 was determined randomly and was found to be a stable scenario. This configuration changes during the early stages but later reaches a stable state. The number of cells or agents in this configuration is 49 since the grid used is 7 × 7.

**Figure 3.**
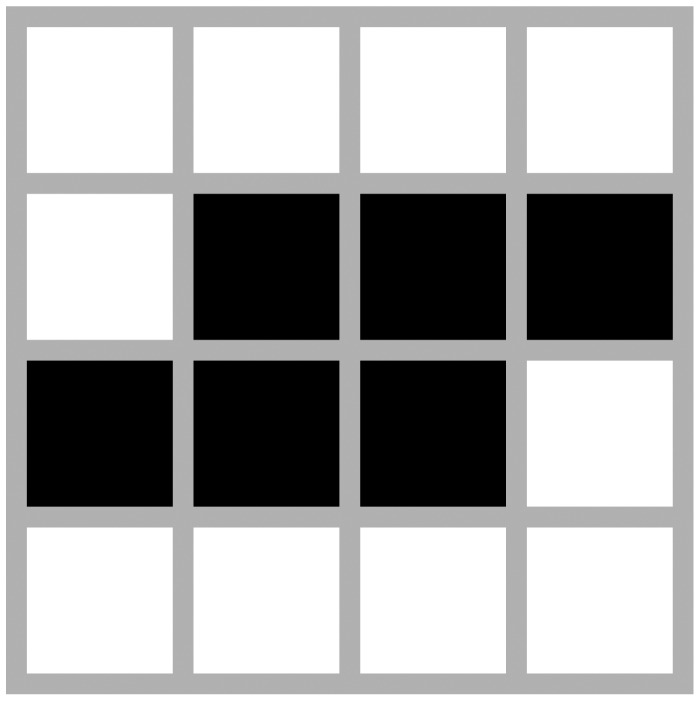
Toad.

The configuration presented in Figure 3 is known as Toad. This oscillator is a bit more complex than the Blinker in terms of the number of agents involved or affected by the oscillation. However it has also a period of 2 like Blinker. This oscillator fits into a grid of 4 × 4, thereby containing 16 agents within the system.

**Figure 4.**
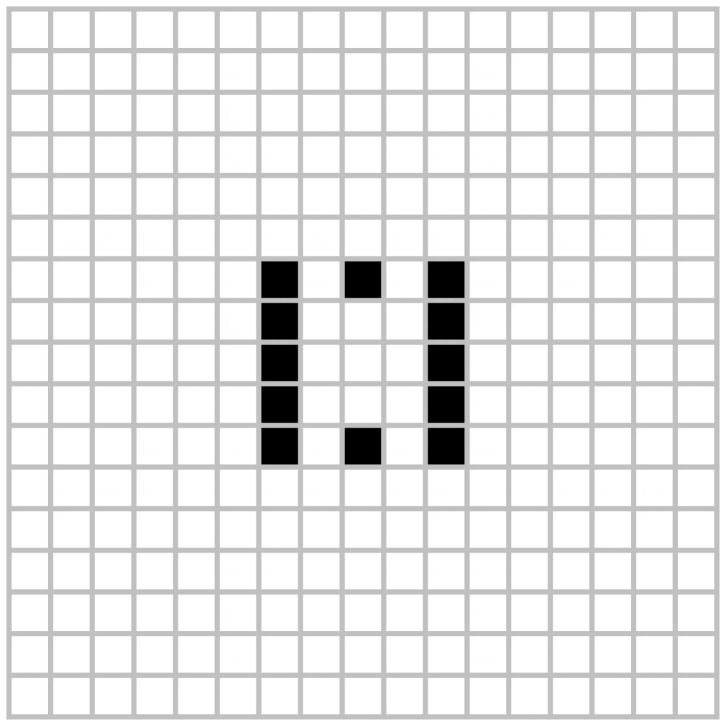
Pulsar.

**Figure 5.**
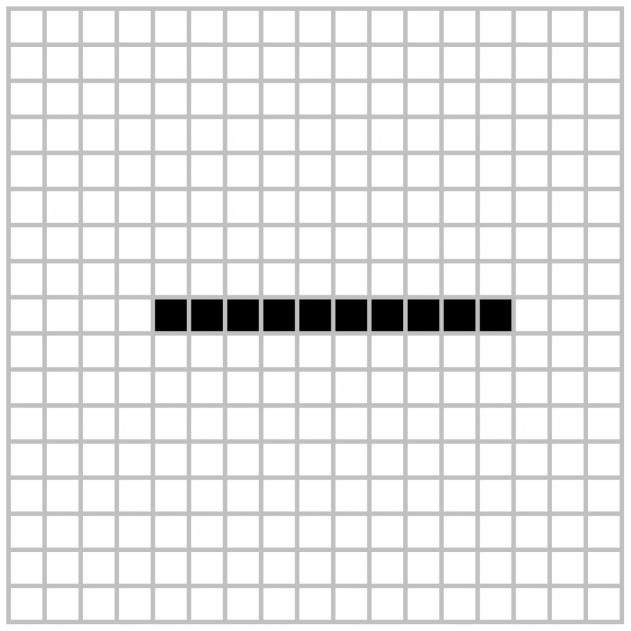
10 cell row.

The previous scenarios can be considered as simple configurations, as intelligent environments can contain a larger number of devices or agents involved, and the evaluation of the system is crucial to determine whether the proposed solution can work with more complex scenarios. In the following examples we will introduce complex configurations called *Pulsar* and *10 Cell Row* shown in Figures 4 and 5. In these configurations there are 289 cells or agents on a 17 × 17 grid, allowing complex behavior and potential oscillations on them.

## 6. Using Optimization Algorithms to Solve the Problem of Cyclic Instability

In order to solve the problem of cyclic instability using different optimization algorithms we need to minimize the amplitude of the oscillations. In the ideal case this would result in a stable system. Additionally we are interested on affecting the fewest number of agents (agents locked).

In order to test these approaches we used the Game of Life because it is a well know system that possesses proven oscillatory behavior in very simple environment with simple agents and few rules. For the test we consider a Game of Life with open boundary conditions. The open boundary condition in our case is considered cold (in terms of the heat entropy) and all cells outside the grid are considered dead. We enriched the game of life with additional conditions: a list of agents that are allowed to be locked. All techniques can lock them according to their results. This is because priority agents (such as alarms, security systems, *etc.*) should not be disabled.

Each solution vector represents the list of blocked agents where the aim is to minimize the *Average Cumulative Oscillation* (ACO) of the system in a given period of time. The ACO is calculated using the following Equation (8) [26].

(8) $$\text{o}=\frac{\sum_{\text{i}=1}^{\text{n}-1}\left|{\text{S}}_{\text{i}}-{\text{S}}_{\text{i}+1}\right|}{\text{n}-1}$$

where *o* is the Average Cumulative Oscillation, *n* is the game of life generations, *S_i_* is the state of the system at the time *i*, *S_i_*_+1_ is the state of the system at the time *i* + 1.

The best solution should not only minimize the amplitude of oscillation but also the number of agents locked. In these experiments the percentage of agents that can be locked is included as a parameter. This is important because as this percentage grows the systems becomes more disabled.

In these experiments we consider systems whose adjacency matrix are of 3 × 3, 4 × 4,7 × 7, and 17 × 17. In all the cases the maximum percentage of locked agents set to 20%.

If an algorithm can not find a better solution in terms of the amplitude of the oscillations, no agents will be locked. If a solution is good in terms of the amplitude of the oscillation but the percentage of locked agents is bigger than the maximum permitted, the solution will be penalized by adding a constant value (in our case the value is 10) to the value of the Average Cumulative Oscillation (ACO).

In our experiments we set a parameter of 3,000 functions calls as the measure of success of the algorithms, *i.e.*, the system has 3,000 opportunities to find a better solution. If after 3,000 functions calls a better solution is not found, the system is deemed to have failed.

## 7. Experimental Results

For the test performed with PSO and BSO for all test instances we used the parameters shown in Table 1.

The parameters used for the *μ*PSO are shown in Table 2.

The parameters used for AIS are shown in Table 3.

The parameters used for GA are shown in Table 4.

For the test performed with MIMIC for all test instances we used the parameters shown in Table 5.

Table 6 shows the level of oscillation obtained for each of the instances taken from the Game of Life. The value obtained assumed that the system remain unchanged during its evolution, *i.e.*, from the initial conditions, the system is allowed to evolve according to the rules of the scenario itself without interference for the entire time of evaluation.

With the parameters described before, we obtain the results shown in Tables 7 and 8. The best result obtained with 100 executions for each test instance is shown.

Table 9 shows the numbers of agents blocked, which correspond to the results obtained with the OAP shown in Tables 7 and 8.

**Figure 6.**
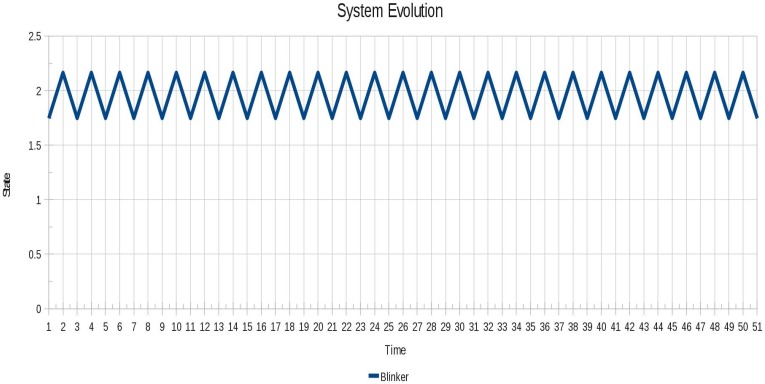
Oscillatory behavior of the instance 1 (Blinker).

**Figure 7.**
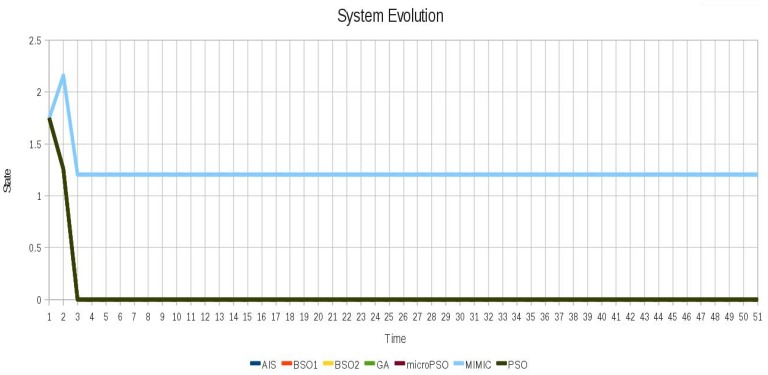
Instabilities are successfully removed for instance 1 (Blinker) using all algorithms.

In order to see the cyclic instability and how it is removed, for each test instance we show the evolution of the system before and after locking. In Figure 6 the oscillatory behavior of instance 1 (Blinker) is shown and in Figure 7 the instabilities are successfully removed from the instance 1 (Blinker). In Figure 7 different evolutions can be seen, because for this scenario different sets of locked agents can achieve system stability.

**Figure 8.**
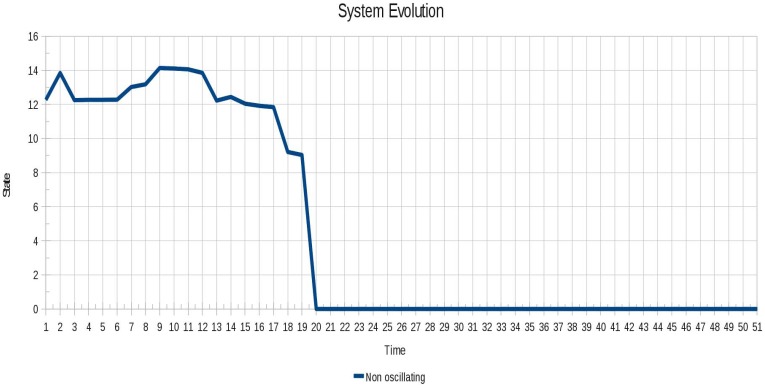
Behavior of the instance 2 (Non-oscillating).

**Figure 9.**
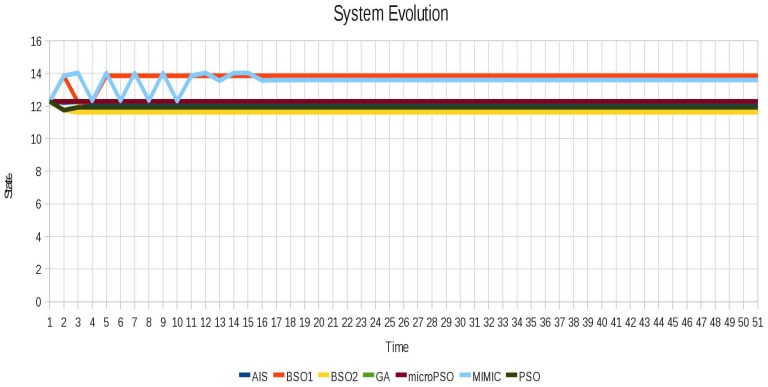
Behavior of the instance 2 (Non-oscillating) after locking with all algorithms.

In the case of instance 2 (Non-oscillating) shown in Figure 8 although the system does not oscillate the techniques were able to find different configurations for this scenario that remain stable while decrease the value of the Average Cumulative Oscillation. The behavior of instance 2 (non-oscillating) after locking is shown in Figure 9.

**Figure 10.**
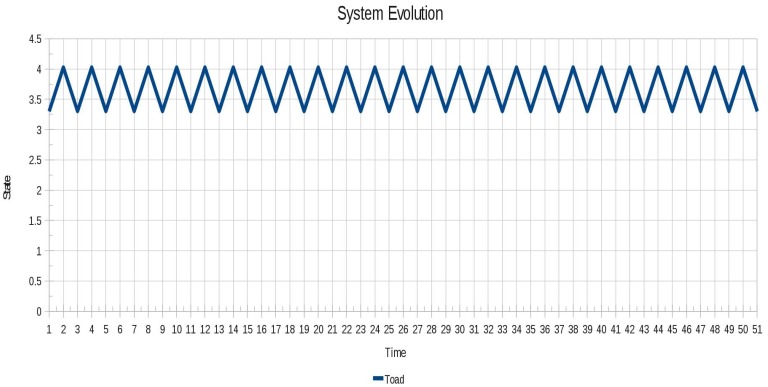
Oscillatory behavior of the instance 3 (Toad).

As for instance 3 (Toad), the oscillatory behavior shown in Figure 10 looks very similar to instance 1 (Blinker).

**Figure 11.**
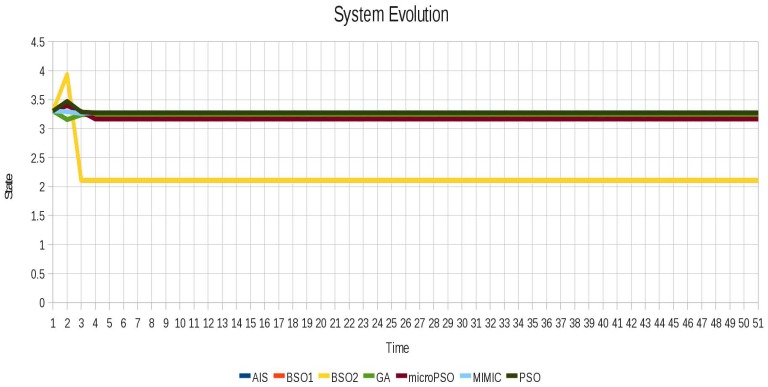
Instabilities are successfully removed for the instance 3 (Toad) using all algorithms.

In the same way as in instance 1(Blinker), instability was eliminated successfully for instance 3 (Toad) as shown in Figure 10. As shown in Tables 7 and 8, different values of the ACO were obtained for this scenario because there are different vectors of locked agents that allow to stabilize the system. This explains why different system evolution is shown in Figure 11 after applying the locking.

**Figure 12.**
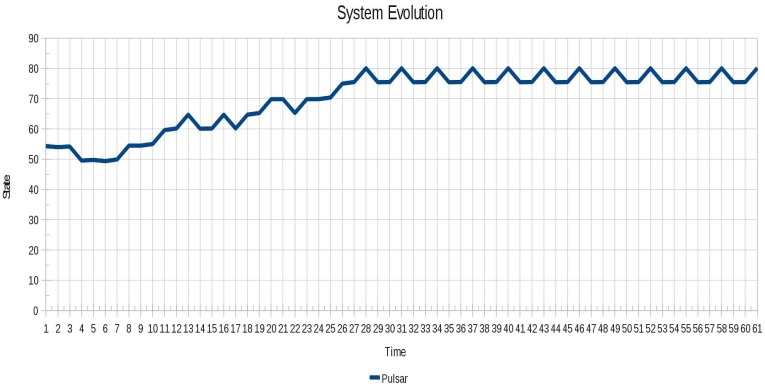
Oscillatory behavior of the instance 4 (Pulsar).

In Figure 12 the oscillatory behavior of instance 4 (Pulsar) is shown, which is a more complex behavior in relation to those shown above.

While in the above instances the best values obtained by the techniques are very similar, this trend is no longer maintained for the instance 5 (Pulsar). The is because the size of the instance considerably increases the number of possible combinations.

**Figure 13.**
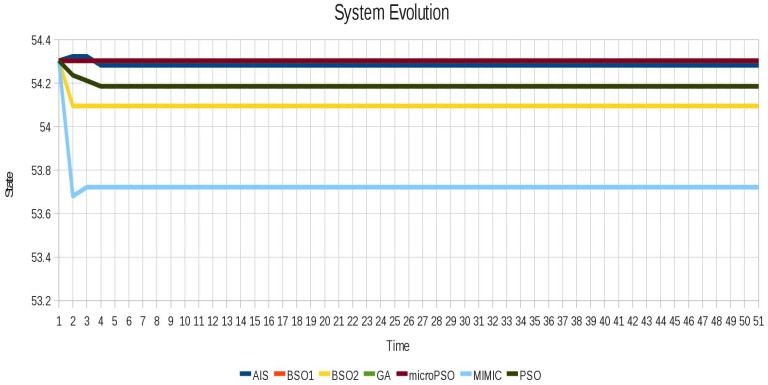
Instabilities are successfully removed for the instance 4 (Pulsar) using all algorithms.

Most importantly, despite the difference in the results between the various techniques, it was possible to stabilize the system with different combinations of locked agents, showing that depending on the scenario there may be more than one set of locked agents with which the system can become stable. This is showcased by the different results obtained for the instance 4 (Pulsar) in the level of instability (refer to Figure 13), where we can see how quickly the system stabilizes in each of the different configurations achieved by the optimization techniques.

**Figure 14.**
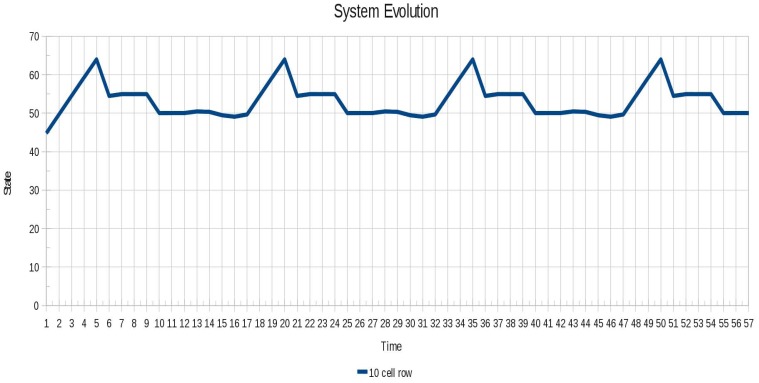
Oscillatory behavior of the instance 5 (10 cell row).

The oscillatory behavior of the instance 5 (10 cell row) is shown in Figure 14. It is seen that the oscillation began quickly in contrast to the Instance 4 (Pulsar), whose oscillation is seen until after a certain period of time.

**Figure 15.**
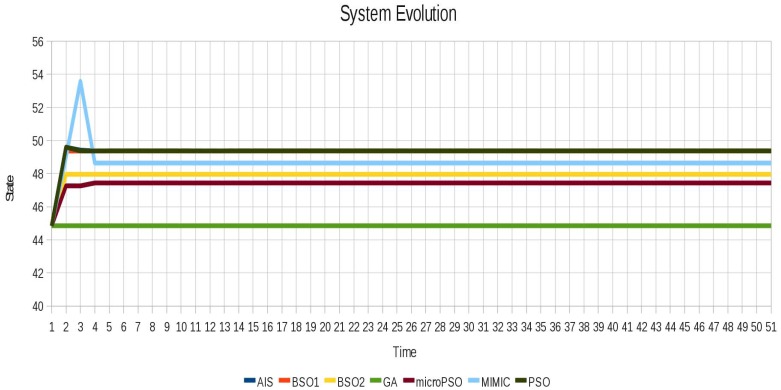
Instabilities are successfully removed for the instance 5 (10 cell row) using all algorithms.

For the instance 5 (10 cell row), again the number of agents represented is therefore significant. The performance results are similar to those described for instance 4 (Toad) and the best results obtained by the techniques vary with respect to each other. The behavior without oscillation is shown in Figure 15. The difference between the behavior of each algorithm responds to the different sets of locked agents by each of the techniques.

Table 10 shows the comparison among the results obtained by different algorithms. To determine whether an algorithm outperforms another, the Wilcoxon signed rank test was performed. The best performing algorithms are those that exceed a greater number of algorithms in the results obtained for the ACO and the number of locked agents.

Despite the similarity of results of some algorithms based on Table 10, it can be said that the GA was able to obtain smaller values relative to the values of the ACO. But if we take into account the number of locked agents, the algorithms PSO and *μ*-PSO are those who achieved best results. This becomes more important because the algorithms PSO and *μ*-PSO achieve system stability, which makes them better by allowing most of the devices continue to work normally.

## 8. Conclusions and Future Work

From our experiments we found that all the algorithms were able to find a vector of locked agents that prevent the system from oscillating. Additionally, Wilcoxon test showed that GA gave better results but not in terms of time and number of agents locked—rather, PSO and *μ*-PSO gave the better results in that sense. MIMIC consistently violated the restriction of maximum permitted percentage of agents locked. MIMIC is based on estimating the distribution of data and for that reason needs a larger number of data (in our case the amount of list of agents locked), and that is the main reason the time spent for the algorithm to find a solution increased significantly.

An important advantage of this proposal compared to others found in the literature is the way by which the system is evaluated, since it only sees the general state of the environment regardless of its topology. Because of this, the evaluation time of the scenarios can be significantly reduced regardless of the number of agents that form part of the system. Additionally, the possibility of a proposal able to work with any scenario can be more clearly distinguished in real time, which helps to improve their operation.

This approach based on the concept of *Average Cumulative Oscillation* opens the possibility for other algorithms to be applied to the problem of cyclic instability, in general algorithms for discrete optimization. In particular we are interested in testing this approach for the case of nomadic and weighted agents and with different percentage of locked agents. Also it is possible to improve the estimation of the oscillation in order to discriminate between stable systems with abrupt changes and systems with small oscillations, because in some cases it is possible to get small values of *Average Cumulative Oscillation* in oscillating systems. We hope to report these results in future.

## Figures and Tables

**Table 1.** Parameters used in PSO and BSO.

**Parameter**	**Value**
Particles	45
*w*	1
*c*_1_	0.3
*c*_2_	0.7

**Table 2.** Parameters of *μ*-PSO.

**Parameter**	**Value**
Particles	6
*w*	1
*c*_1_	0.3
*c*_2_	0.7
Replacement generation	100
Number of restart particles	2
Mutation Rate	0.1

**Table 3.** Parameters of AIS.

**Parameter**	**Value**
Antibodies	45
Antibodies to select	20
New Antibodies	20
Factor Beta	2

**Table 4.** Parameters of GA.

**Parameter**	**Value**
Chromosomes	45
Mutation percentage	0.15
Elitism	0.2
Clones percentage	0.3
Scouts percentage	0.8

**Table 5.** Parameters used in MIMIC.

**Parameter**	**Value**
Individuals	100
Elitism	0.5

**Table 6.** Instance.

**Instance**	**Matrix**	**# of Agents**	***S*_0_**	***S****_f_*	**ACO**
1 (Blinker)	3 × 3	9	1.748188027	1.7481880270062005	0.416164
2 (Non-oscillating)	7 × 7	49	12.284241189	0.0	0.196199
3 (Toad)	4 × 4	16	3.6261349786353887	3.6261349786353887	0.974857
4 (Pulsar)	17 × 17	289	54.30350121388617	75.43697161100698	2.916489
5 (10 cell row)	17 × 17	289	44.85304503100554	50.04149156221928	2.23957

**Table 7.** ACO Results (A).

**Instance**	**Average Cumulative Oscillation**			

	PSO	MIMIC	BSO1	BSO2
1 (Blinker)	0.0173	0.4161648288	0.0173	0.0173
2 (Non-oscillating)	0.0036684768	10.0040328723	0.0013180907	6.96E–4
3 (Toad)	0.0027436818	10.0005786421	0.0031827703	0.0027436818
4 (Pulsar)	0.0039470374	10.000434825	0	0
5 (10 cell row)	0.0461757189	10.040107898	1.58E–7	0

**Table 8.** ACO Results (B).

**Instance**	**Average**	**Cumulative**	**Oscillation**

	AIS	GA	*μ*PSO
1 (Blinker)	0.0173	0.0173	0.0173
2 (Non-oscillating)	0.00239	4.77E–6	4.77E–6
3 (Toad)	0.00253	0.00253	0.0025307973
4 (Pulsar)	6.12E–4	5.24E–13	4.3E–4
5 (10 cell row)	0.0472	1.60E–10	0.0256051082

**Table 9.** Locked Agents.

**Instance**	**# of Locked Agents**							

	Allow	PSO	BSO1	BSO2	AIS	GA	*μ*-PSO	MIMIC
1 (Blinker)	1	1	1	1	1	1	1	0
2 (Non-oscillating)	9	1	9	8	4	12	8	19
3 (Toad)	5	3	3	3	3	3	3	9
4 (Pulsar)	57	26	43	56	31	53	28	141
5 (10 cell row)	57	31	38	44	33	57	18	139

**Table 10.** Comparison among algorithms based on Wilcoxon test.

**Algorithm**	**Number of Algorithms**			

	by ACO Value		by # of locked Agents	

	Overcome	Not Overcome	Overcome	Not Overcome
PSO	1	5	5	1
BSO1	2	4	3	3
BSO2	3	3	3	3
*μ*PSO	5	1	6	0
AIS	4	2	4	2
GA	6	0	1	5
MIMIC	0	6	0	6
